# Handgrip Strength Is Inversely Associated With the Progression of Kidney Damage in a General Japanese Population: A Prospective Cohort Study

**DOI:** 10.7759/cureus.71276

**Published:** 2024-10-11

**Authors:** Shiqi Deng, Urme Binte Sayeed, Yukiko Wagatsuma

**Affiliations:** 1 Department of Clinical Trial and Clinical Epidemiology, Graduate School of Comprehensive Human Sciences, University of Tsukuba, Tsukuba, JPN; 2 Department of Clinical Trial and Clinical Epidemiology, Institute of Medicine, University of Tsukuba, Tsukuba, JPN

**Keywords:** general population, handgrip strength, kidney disease, kidney function, muscle strength

## Abstract

Background

Handgrip strength is an indicator of muscle function and a predictor of health outcomes. However, only a few studies have examined the association between handgrip strength and the development of kidney damage. This study aimed to investigate the longitudinal association of handgrip strength with kidney damage in a general Japanese population.

Methods

This prospective cohort study enrolled participants with normal kidney function who attended annual health check-ups in Ibaraki Prefecture, Japan, between April 2016 and March 2020. Clinical information, including data from blood and urine tests, physiological examinations, and handgrip strength tests, was collected at enrollment. Lifestyle information was also collected via a self-administered questionnaire. The study participants were followed up for the progression of kidney damage until March 2023. Relative handgrip strength was calculated by dividing the handgrip strength by the body mass index to adjust for differences in body mass. A Cox proportional hazards model was used to examine the relationship between relative handgrip strength and the progression of kidney damage.

Results

A total of 4304 participants with normal kidney function were enrolled in this study. During the mean follow-up period of approximately 4 years (SD 1.8 years), 15.4% of the participants developed kidney damage. After adjusting for covariates, higher relative handgrip strength was associated with a lower risk of kidney damage in men (HR = 0.63, 95% CI: 0.43 - 0.90; p = 0.012), but no significant association was observed in women.

Conclusions

Higher relative handgrip strength is associated with a lower risk of kidney damage in men. This finding highlights the importance of muscle strength in preventing kidney damage.

## Introduction

The increasing number of chronic kidney disease (CKD) cases is an important global health issue. End-stage renal disease (ESRD) and cardiovascular complications frequently occur in CKD patients, leading to a high overall mortality rate [1]. In Japan, the estimated prevalence of CKD is 14.6% [2]. Additionally, renal function decreases with age, making the aging population a contributing factor to the rise in CKD incidence [3]. As Japan's population continues to age, the ongoing challenge of preventing the progression of kidney damage remains. If CKD is left untreated, it can progress to kidney failure, ultimately necessitating dialysis due to ESRD. Conversely, it is possible to prevent the onset of CKD through lifestyle modifications and appropriate early intervention [4].

Handgrip strength is a widely used method to measure muscle strength because it is simple, inexpensive, and reliable [5]. It has been reported that lower handgrip strength is associated with a greater incidence of physical function decline, cardiovascular disease, and overall mortality [5,6]. Previous studies have demonstrated a cross-sectional relationship between handgrip strength and CKD, with findings indicating that handgrip strength is inversely associated with the existence of CKD [7]. In addition, higher handgrip strength is associated with a lower risk of all-cause mortality in patients with CKD [8]. However, those studies focused on handgrip strength as an indicator of outcomes in advanced CKD patients. Few studies have examined the relationship between handgrip strength and the development of early-stage CKD [9,10], and evidence of the effect of handgrip strength on CKD progression is scarce in the Japanese population.

The Ministry of Health, Labour, and Welfare of Japan recommends that people who are at a moderately increased risk of developing CKD according to their estimated glomerular filtration rate (eGFR) and development of albuminuria be given appropriate health guidance. These relatively early stages of kidney damage are present in 8.86 million people in Japan [11], accounting for two-thirds of the total number of people with CKD. If the association between handgrip strength and relatively early-stage kidney damage can be clarified, it can be utilized for health guidance on improved muscle function and CKD prevention.

Studies have noted that relative handgrip strength, calculated as handgrip strength divided by body mass index (BMI), is a more reliable indicator for assessing metabolic disorders and cardiovascular health than handgrip strength itself [12-14]. In studies related to muscle function, BMI has been suggested to regulate muscle strength. BMI-adjusted relative handgrip strength was used as an indicator to adjust for differences in body mass.

Given the limited information on the relationship between relative handgrip strength and the progression of kidney damage, this study aims to investigate the association of relative handgrip strength with kidney damage in a general Japanese population. We hypothesized that persons with higher relative handgrip strength have a lower risk of kidney damage progression over time.

## Materials and methods

Study participants

Study participants were enrolled in an ongoing cohort study conducted at a regional healthcare center in Mito and its outreach service sites in Ibaraki Prefecture, Japan. The cohort initially included 9,000 participants, with approximately 5,000 subjects regularly participating in annual health check-ups.

This prospective cohort study included individuals with normal kidney function who participated in health check-ups between April 2016 and March 2020. The baseline time point was defined as the date of the first enrollment for each participant within this time period. Baseline data were collected on demographic characteristics, anthropometric measurements, clinical indicators, lifestyle factors, and handgrip strength. The studies excluded those who (1) were aged younger than 18 years, (2) were missing handgrip strength measurements, or (3) were missing data on eGFR or proteinuria. After enrollment, follow-ups were conducted until March 31, 2023. The participants were observed until the onset of kidney damage or the endpoint of the study. The number of follow-ups was calculated from the date of the first health check-up to the date when participants were observed to have an incident of kidney damage, or 31 March 2023, which occurred first. Those who did not experience kidney damage were defined as right-censor data at the end of observation.

**Figure 1:**
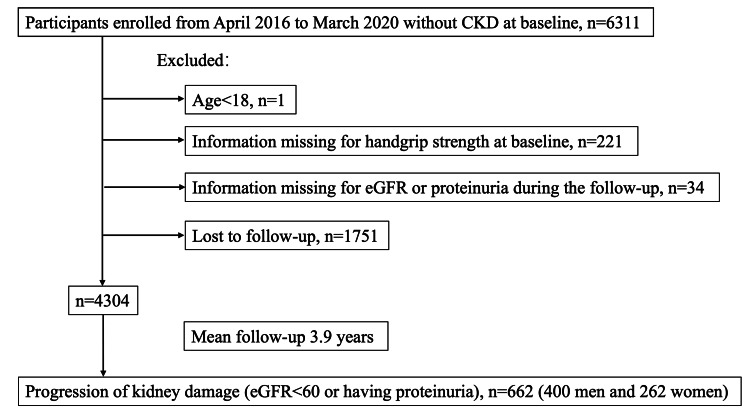
Study flow chart.

In this study, 6311 participants with no record of kidney damage were enrolled from April 2016 to March 2020. In total, 2007 participants were excluded from the study because they were under 18 years old (n = 1), had missing data for handgrip strength (n = 221), had missing data for eGFR or proteinuria during the follow-up (n = 34), or were lost to follow-up (n = 1751). There were 4304 participants included in the final analysis (Figure 1).

Measurements

Anthropometric measurements of height and weight were taken using a TANITA DC250 total body composition analyzer (TANITA Co. Ltd., Japan), and the corresponding BMI was calculated. Systolic blood pressure, diastolic blood pressure, and waist circumference were measured by medical staff at regional hospitals or outreach sites. Proteinuria was measured using a urine protein dipstick. Fasting blood samples were collected after the individuals had fasted overnight. Biochemical indicators, including fasting plasma glucose levels, glycated hemoglobin (HbA1c) levels, low-density lipoprotein levels, high-density lipoprotein levels, triglyceride levels, and eGFR, were measured in the hospital laboratory. A standardized self-administered questionnaire provided by the Japan Ministry of Health, Labour, and Welfare was used to collect lifestyle-related data as well as medical history [15]. In the present study, questions about lifestyle and medical history related to kidney function, including smoking, exercise frequency, alcohol consumption, daily walking, eating snacks, skipping breakfast, adequate sleep, and the use of antihypertensive, anticholestatic, and antidiabetic agents, were selected. The questionnaire is presented in the Appendices.

Handgrip strength

Handgrip strength was tested using a Smedley digital handgrip dynamometer (TAKEI Corporation, Japan) [13]. Trained personnel conducted all measurements following a standardized protocol to ensure uniformity, and the dynamometer was calibrated before each session. The hand dynamometer was preadjusted to optimize the participants' force exertion. Participants performed the test in a standing position, with their arms naturally placed at the sides. Each hand was tested twice, with participants instructed to squeeze the dynamometer as hard as possible for 3 seconds. The first round of measurements was taken for the right and left hands consecutively, followed by a second round of measurements in the same order. The mean of the maximum value for each hand was used to indicate the handgrip strength. Relative handgrip strength was calculated by dividing handgrip strength by BMI to adjust for differences in body mass [13].

Definition

Kidney damage was defined as an eGFR < 60 ml/min/1.73 m^2^ and/or a positive result for proteinuria (≥ 1+, 30 mg/dL). The eGFR was calculated from the serum creatinine concentration using the conversion formula for the Japanese population, which was prepared by the Japanese Society of Nephrology [16]. BMI (kg/m^2^) was categorized as < 18.5 (underweight), 18.5 to 24.9 (normal), 25.0 to 29.9 (overweight), and ≥ 30 (obese). Hypertension was defined as having an SBP ≥ 140 mmHg and/or a DBP ≥ 90 mmHg and/or being prescribed medications for hypertension [17]. Diabetes mellitus was defined as having a HbA1c level ≥ 6.5 mmol/mol and/or fasting blood glucose ≥ 126 mg/dl and/or being prescribed antidiabetic medications [18]. Dyslipidemia was defined as a low-density lipoprotein level ≥ 140 mg/dL, a high-density lipoprotein level < 40 mg/dL, a triglyceride level ≥ 150 mg/dL, and/or the use of anti-cholestatic medications [19].

Statistical analyses

The averages and standard deviations (SDs) for continuous variables or the numbers with percentages for categorical variables were used to express baseline characteristics. The ANOVA test and chi-square test were used to compare continuous and categorical variables, respectively. The Cox proportional hazards model was applied to assess the relationship between relative handgrip strength (as a continuous variable) and the progression of kidney damage. The time to incident kidney damage was calculated from the date of baseline health check-up to the date of the kidney damage event or the end of follow-up. Hazard ratios (HRs) and 95% confidence intervals (CIs) were calculated to represent the risk of kidney damage for each unit increase in relative handgrip strength. Afterward, sex stratification analysis was performed. Model 1 was adjusted for age and baseline eGFR. Model 2 was adjusted for the variables in Model 1 and comorbidities (hypertension, diabetes, and dyslipidemia). Model 3 was adjusted for the variables in Model 2 and lifestyle behaviors (smoking, regular exercise, alcohol consumption, daily walking, eating snacks, skipping breakfast, and adequate sleep). As a secondary analysis, handgrip strength was also investigated in association with kidney damage using the same Cox proportional hazards model applied in the primary analysis. The analysis was performed using IBM SPSS Statistics for Windows, Version 29.0.2.0 Armonk, NY: IBM Corp. P-values < 0.05 were considered statistically significant.

Ethics approval and consent to participate

This study was approved by the Research Ethics Committee of the Faculty of Medicine at the University of Tsukuba (approval number: 1000). The study was conducted in accordance with the Declaration of Helsinki. The study subjects provided written informed consent to participate.

## Results

There were 4304 participants included in the study. Kidney damage occurred in 262 (13.4%) women and 400 (17.0%) men over an average follow-up period of 3.9 years (SD 1.8 years). The baseline characteristics of the participants are shown in Table 1. The average age was 48.7 years (SD 14.3 years). Compared with men, women were older at baseline. The highest proportion of the subjects were between the ages of 40 and 59 years (47.6%) and within the normal BMI group (63.3%). A larger proportion of underweight participants were women than men, whereas the proportion of overweight and obese participants was lower among women than men. The baseline characteristics of the cohort and excluded participants are shown in the Appendices.

**Table 1:** Baseline characteristics of study participants. Continuous variables are presented by mean ± SD, categorical variables are presented by the number (%). Missing, *1, n=46; *2, n=50; *3, n=59; *4, n=58; *5, n=60; *6, n=57; *7, n=88. p-value by ANOVA test and chi-square.

	All	Men	Women	*p-*value
	n=4304	n=2348	n=1956	
Age (years)	48.7 ± 14.3	47.2 ± 14.4	50.4 ± 13.9	<0.001
Age group				<0.001
18-39	1176 (27.3%)	738 (31.4%)	438 (22.4%)	
40-59	2048 (47.6%)	1078 (45.9%)	970 (49.6%)	
60-93	1080 (25.1%)	532 (22.7%)	548 (28.0%)	
BMI (kg/m^2^)	23.5 ± 3.8	24.3 ± 3.7	22.6 ± 3.8	<0.001
Underweight	261 (6.1%)	61 (2.6%)	200 (10.2%)	
Normal	2725 (63.3%)	1417 (60.3%)	1308 (66.9)	
Overweight	1074 (24.9%)	706 (30.1%)	368 (18.8%)	
Obese	244 (5.7%)	164 (7.0%)	80 (4.1%)	
eGFR (mL/min/1.73 m^2^)	79.6 ± 13.1	79.6 ± 12.6	79.6 ± 13.7	0.939
Hypertension				<0.001
Yes	1534 (35.6%)	939 (40.0%)	595 (30.4%)	
No	2770 (64.4%)	1409 (60.0%)	1361 (69.6%)	
Dyslipidemia				<0.001
Yes	1954 (45.4%)	1154 (49.1%)	800 (40.9%)	
No	2350 (54.6%)	1194 (50.9%)	1156 (59.1%)	
Diabetes				<0.001
Yes	336 (7.8%)	238 (10.1%)	98 (5.0%)	
No	3968 (92.2%)	2110 (89.9%)	1858 (95.0%)	
Current Smoking^*1^				<0.001
Yes	967 (22.7%)	775 (33.4%)	192 (9.9%)	
No	3291 (77.3%)	1543 (66.6%)	1748 (90.1%)	
Regular Exercise^*2^				<0.001
Yes	883 (20.8%)	551 (23.8%)	332 (17.1%)	
No	3371 (80.2%)	1765 (76.2%)	1606 (82.9%)	
Alcohol Drinking^*3^				<0.001
Daily	917 (21.6%)	744 (32.2%)	173 (9.0%)	
Occasional and Rarely	3328 (78.4%)	1568 (67.8%)	1760 (91%)	
Daily Walking^*4^				0.016
Yes	1415 (33.3%)	804 (34.8%)	611 (31.6%)	
No	2831 (66.7%)	1509 (65.2%)	1322 (68.4%)	
Eating Snacks^*5^				<0.001
Yes	1133 (26.7%)	568 (24.6%)	565 (29.2%)	
No	3111 (73.3%)	1742 (75.4%)	1369 (70.8%)	
Skipping Breakfast^*6^				<0.001
Yes	795 (18.8%)	524 (22.6%)	271 (14.0%)	
No	3452 (81.2%)	1790 (77.4%)	1662 (86.0%)	
Adequate Sleep^*7^				<0.001
Yes	2780 (65.9%)	1586 (69.2%)	1194 (62.1%)	
No	1436 (34.1%)	707 (30.8%)	729 (37.9%)	

**Figure 2:**
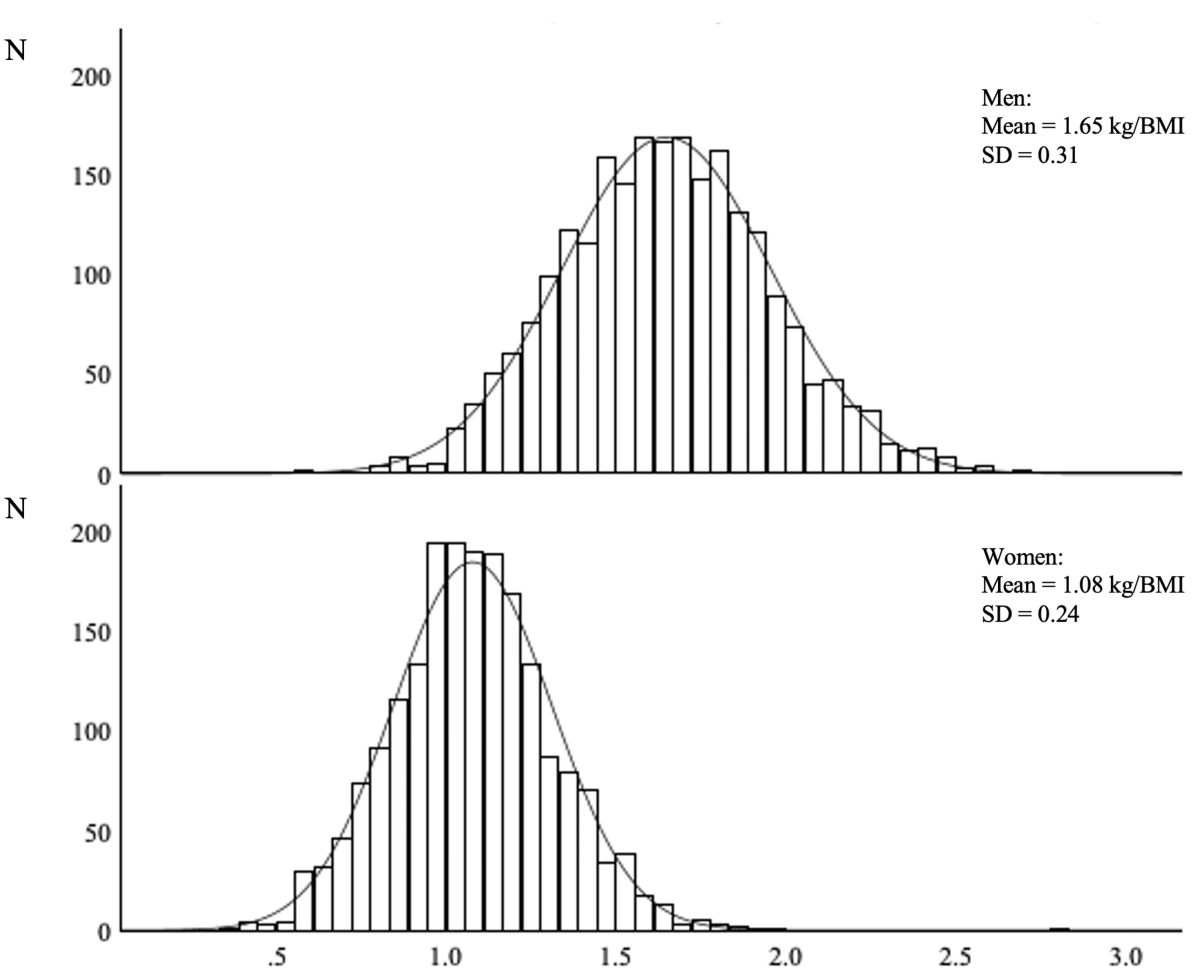
Distribution of relative handgrip strength by sex stratification.

The mean baseline eGFR was 79.6 mL/min/1.73 m^2^, representing a mild decrease in kidney function. There was no significant difference in baseline eGFR between men and women. More men had hypertension, dyslipidemia, and diabetes than women. Additionally, in terms of lifestyle, higher proportions of men smoked, consumed alcohol, and skipped breakfast. However, men were also more engaged in regular exercise and daily walking than women were, avoided eating snacks, and obtained adequate sleep. The mean relative handgrip strength was 1.65 ± 0.31 kg/BMI in men and 1.08 ± 0.24 kg/BMI in women. The distributions of relative handgrip strength by sex are shown in Figure 2.

The results of the Cox proportional hazard model for the progression of kidney damage and relative handgrip strength among all participants are presented in Table 2. In the analysis, after evaluating the interactions between relative handgrip strength and all covariates, no significant interactions were detected. An inverse association was found between relative handgrip strength and the progression of kidney damage (HR = 0.64, 95% CI: 0.48 - 0.88; p = 0.006).

**Table 2:** Cox proportional hazard model for the progression of kidney damage with relative handgrip strength. Model 1: adjusted for sex, age, and eGFR. Model 2: adjusted for the factors of model 1, hypertension, diabetes, and dyslipidemia. Model 3: adjusted for the factors of model 2, smoking, regular exercise, alcohol consumption, daily walking, eating snacks, skipping breakfast, and adequate sleep.

	Hazard Ratio	95% CI		*p-*value
Crude Model	0.90	0.80	1.17	0.730
Model 1 – sex, age and eGFR	0.58	0.44	0.78	<0.001
Model 2 – Model 1 + comorbidities	0.64	0.48	0.87	0.004
Model 3 – Model 2 + lifestyles	0.64	0.48	0.88	0.006

The sex stratification results of the Cox proportional hazard model for the progression of kidney damage are shown in Table 3. For men, the crude model revealed that relative handgrip strength was significantly associated with the progression of kidney damage (p = 0.004). The association remained significant in the final model, with adjustments for age, baseline eGFR, hypertension, dyslipidemia, diabetes, smoking, regular exercise, alcohol consumption, daily walking, skipping breakfast, eating snacks, and adequate sleep (HR = 0.63, 95% CI: 0.43 - 0.90; p = 0.012). For women, the p-values were slightly greater than 0.05 in the crude models and Model 1. However, the associations were not statistically significant after further adjusting for comorbidities and lifestyles.

**Table 3:** Cox proportional hazard model for the progression of kidney damage with relative handgrip strength by sex. Model 1: adjusted for age and eGFR. Model 2: adjusted for the factors of model 1, hypertension, diabetes, and dyslipidemia. Model 3: adjusted for the factors of model 2, smoking, regular exercise, alcohol consumption, daily walking, eating snacks, skipping breakfast, and adequate sleep.

Men					Women				
	Hazard Ratio	95% CI		*p-*value		Hazard Ratio	95% CI		*p-*value
Crude Model	0.62	0.45	0.86	0.004	Crude Model	0.63	0.37	1.07	0.088
Model 1 – age and eGFR	0.59	0.42	0.83	0.002	Model 1 – age and eGFR	0.58	0.33	1.01	0.055
Model 2 – Model 1 + comorbidities	0.64	0.45	0.91	0.014	Model 2 – Model 1 + comorbidities	0.68	0.38	1.20	0.185
Model 3 – Model 2 + lifestyles	0.63	0.43	0.90	0.012	Model 3 – Model 2 + lifestyles	0.76	0.42	1.35	0.344

In the sex-stratified Cox analysis of handgrip strength and kidney damage, handgrip strength was not significant in any of the models. However, it is noteworthy that in the crude model, handgrip strength exhibited a positive association with kidney damage, which shifted to a negative association after adjusting for BMI. This result is presented in the Appendices.

## Discussion

This longitudinal study investigated the association between relative handgrip strength and the risk of kidney damage among a general population participating in health check-ups. Over a follow-up period of 3.9 years, 15.4% of the participants developed kidney damage. Our findings revealed that higher relative handgrip strength was associated with a reduced risk of kidney damage among men but not among women.

This study supports previous research, indicating that greater handgrip strength is associated with a lower risk of kidney damage. To date, only a few studies have explored the association between handgrip strength and kidney damage among the general population. A study conducted in China reported that higher handgrip strength was associated with a lower prevalence of kidney function decline in a community-dwelling Chinese population aged 50 years and above [20]. Another study conducted among a Korean population also reached the same conclusion, with a wider age range (age > 18 years) [7]. However, these studies had a cross-sectional design and provided limited information on the longitudinal relationship between handgrip strength and the progression of kidney damage. One study reported that handgrip strength was negatively associated with the incidence of CKD using data from the UK Biobank population [9]. Another study reported that relative handgrip strength was independently and inversely associated with the incidence of CKD in both men and women in middle-aged to older Korean populations [10]. Our study also suggested that a lower relative handgrip strength was associated with the progression of kidney damage in a healthy population.

There are plausible physiological mechanisms for the observed association, even though the underlying process is still not entirely understood. Handgrip strength is related to systemic factors such as inflammation and insulin resistance. IL-6 and tumor necrosis factor-α (TNF-α) levels are negatively correlated with handgrip strength, and a lower level of inflammation is believed to protect renal function [21]. Increased muscle strength has been shown to increase the protein levels of glucose transporter (GLUT-4), thereby promoting insulin resistance [22]. In addition, contractile skeletal muscle secretes various myokines, such as IL-6, which play a role in regulating glucose metabolism [23]. Under conditions of insulin resistance, insulin influences multiple aspects of kidney function, such as renal hemodynamics, podocyte health, and tubular activity [24]. Sarcopenia, characterized by the loss of skeletal muscle mass and strength, may exacerbate these metabolic and inflammatory changes [25]. Lower handgrip strength, a reflection of reduced muscle function, is a hallmark of sarcopenia and may indicate a decline in overall physical health. This weakened muscle state, combined with metabolic dysregulation, is often observed in CKD patients and may contribute to the progression of kidney damage through both direct metabolic effects and chronic inflammation [26].

This study revealed a counterintuitive association between handgrip strength and kidney damage, with higher handgrip strength linked to an increased risk of kidney damage in the crude model, although this result was not statistically significant. This finding contrasts with results obtained using relative handgrip strength. A plausible explanation for this discrepancy is that individuals with higher body weights tend to exhibit greater handgrip strength, and being overweight is a recognized risk factor for kidney damage. In this cohort, 37.1% of men and 22.9% of women were overweight, potentially confounding the proper relationship between handgrip strength and kidney damage. After further adjustment for BMI, the counterintuitive relationship between handgrip strength and kidney damage was reversed, supporting our hypothesis. The inclusion of both handgrip strength and BMI as variables in the same model primarily tests the independent effects of these variables on the outcome. Using a joint indicator may better explain the contributions of strength and body composition to health outcomes. This result, along with those of previous studies, supports the notion that relative handgrip strength is a more reasonable indicator for assessing health outcomes than handgrip strength [12-14].

Our results revealed an association between handgrip strength and kidney damage in men but not in women after sex stratification. This association persisted across all adjusted models for men but not for women. These sex-specific differences may be attributed to several factors, including variations in muscle physiology, sex hormones, and the level of urinary protein. A previous study assessing the relationships between daily life functions and skeletal muscle mass and strength in Japanese participants reported that handgrip strength is consistently greater in men than in women across all age groups and that the extent of decline with age is greater in men [27]. The sex differences observed in CKD epidemiological data suggest that sex hormones, particularly estrogen, may play a protective role [28]. Estrogen is thought to have anti-inflammatory and antioxidative properties that contribute to the protection of kidney function [29, 30]. Additionally, it may reduce the progression of renal injury by modulating the renin-angiotensin-aldosterone system, decreasing fibrosis, and promoting vasodilation [31]. In an animal study, male 5/6 nephrectomy rats presented signs of malnourishment, anemia, and mild renal damage, whereas the females presented no signs of malnourishment, anemia, or albuminuria. These findings elucidate the molecular pathways involved in the sex-specific pathogenesis of renal injury [32]. Sex differences in the progression of CKD have also been reported. Longitudinal studies have shown that women have a significantly lower risk of progression to ESRD than men do, even after various lifestyles and medical management strategies are considered [33]. Considering that our follow-up period was relatively shorter than that of previous longitudinal studies on handgrip strength and kidney function, the varied effects of baseline handgrip strength may also have manifested in women over a longer period. Furthermore, previous studies have reported a significant interaction between sex and proteinuria; the main independent predictive risk factor for a rapid decline in the eGFR in men was proteinuria, whereas this interaction was not detected in women [34]. In this study, proteinuria was included as an indicator of kidney damage, which may have influenced the results. High levels of urinary protein are related to unhealthy lifestyles and obesity, which are also risk factors for lifestyle-related diseases such as diabetes. The proportion of individuals with obesity, unhealthy lifestyle habits, and lifestyle-related diseases was greater in men than in women. Therefore, differences in lifestyle may have affected the results. The differences in kidney damage risk between men and women need to be investigated further.

As an easy-to-use and objective tool, handgrip strength may serve as a potential indicator for screening and identifying individuals at high risk for future kidney damage. Regular handgrip strength assessments may allow for earlier detection of kidney damage, enabling timely interventions that could slow disease progression. Consequently, community populations can be encouraged to engage in physical activity to maintain or even enhance muscle function. Incorporating handgrip strength measurements into annual health examinations offers a simple and effective way to monitor overall health and detect changes in muscle function over time, providing an early warning sign for potential kidney damage.

The strengths of this study include the relatively large number of participants with stable annual follow-up, allowing for the accurate observation of longitudinal trends in kidney function. Additionally, the wide age range of the individuals attending the annual medical examinations enhances the generalizability of the findings across different age groups. Furthermore, the prospective cohort design enables the temporal relationship between handgrip strength and kidney damage to be established, contributing to the robustness of the findings.

Limitations

There are several limitations to consider. First, the information came from an annual health check-up with a single test for blood and urine samples. This approach may lead to the inclusion of participants who do not have persistent morphological or organic kidney damage. Second, generalizability might be compromised because our study population consists of individuals who participated in an annual health check-up at a regional hospital, which may indicate greater health consciousness than the general population has. Participants who regularly attend health check-ups are more likely to engage in healthier behaviors, such as better diet and regular exercise, which could affect kidney function outcomes. Consequently, our findings may underestimate the true incidence of kidney damage in a less health-conscious population. Third, significant variations were observed in the baseline characteristics, such as age, eGFR, and blood glucose levels, between the excluded and included populations. Although these differences may impact the results, these clinical biomarkers naturally increase with age. Furthermore, the study design and analytical methods employed in this research adequately controlled for these clinical biomarkers. Therefore, we believe that these differences are unlikely to have a substantial effect on the outcomes.

Further studies are needed to extend the follow-up period to better understand the long-term effects of handgrip strength on kidney health. Additionally, conducting studies in more geographically diverse populations would enhance the generalizability of the findings. 

## Conclusions

Higher relative handgrip strength was associated with a lower incidence of kidney damage after approximately four years of follow-up in men. Our findings highlight the importance of high muscle strength in preventing kidney damage. Encouraging people to maintain muscle strength would be an effective way to prevent kidney damage.

## Appendices

**Table 4:** Questionnaire.

Questions	Answer
Are you currently using an anti-hypertensive drug?	□Yes □No
Are you currently using insulin injection or an antidiabetic (hypoglycemic) drug?	□Yes □No
Are you currently using an anti-cholesteric agent?	□Yes □No
Have you ever been diagnosed as having chronic renal failure by a physician, or got artificial dialysis?	□Yes □No
Have you smoked in the last month?	□Yes □No
Have you exercised more than 30 minutes per 1 time, more than 2 times per week, more than 1 year?	□Yes □No
Do you walk daily or do other physical activity equal to walking more than 1 hour per day?	□Yes □No
Do you have a snack (night meal) after dinner more than 3 times a week?	□Yes □No
Do you skip breakfast more than 3 times a week?	□Yes □No
How often do you drink alcohol (such as sake, shochu, beer, whisky etc.)?	□Everyday □Sometimes □None
Do you sleep enough?	□Yes □No

**Table 5:** Baseline characteristics of cohort and excluded participants.

	Excluded n=2007	Cohort n=4304	*p-*value
Age	51.7 ± 14.6	48.7 ± 14.3	<0.001
Sex			<0.001
Men	1006 (50.1%)	2348 (54.6%)	
Women	1001 (49.9%)	1956 (45.4%)	
BMI	23.4 ± 3.8	23.5 ± 3.8	0.200
eGFR (mL/min/1.73 m^2^)	78.7 ± 12.7	79.6 ± 13.2	0.011
Systolic blood pressure	127.0 ± 18.9	128.5 ± 18.9	0.003
Diastolic blood pressure	77.3 ± 12.8	77.3 ± 12.8	0.207
HbA1c (%)	5.76 ± 0.59	5.71 ± 0.64	0.002
Fasting Plasma Glucose (mg/dL)	102.6 ± 18.8	101.0 ± 20.1	0.004
High-density lipoprotein cholesterol	60.9 ± 15.4	60.0 ± 14.9	0.025
Low-density lipoprotein cholesterol	120.0 ± 31.8	120.9 ± 31.6	0.285
Triglyceride	105.7 ± 76.7	106.9 ± 83.6	0.594

**Table 6:** Cox proportional hazard model of progression of kidney damage and absolute handgrip strength stratified by sex. Model 1: adjusted for BMI. Model 2: adjusted for the factors of model 1, age and eGFR. Model 3: adjusted for the factors of model 2, hypertension, diabetes, dyslipidemia, smoking, regular exercise, alcohol consumption, daily walking, eating snacks, skipping breakfast, and adequate sleep.

Men					Women				
	Hazard Ratio	95% CI		*p*-value		Hazard Ratio	95% CI		*p-*value
Crude Model	1.00	0.99	1.02	0.831	Crude Model	1.02	0.99	1.05	0.207
Model 1	0.99	0.98	1.01	0.522	Model 1	1.01	0.98	1.04	0.461
Model 2	0.99	0.97	1.00	0.176	Model 2	0.99	0.96	1.02	0.698
Model 3	0.99	0.97	1.00	0.120	Model 3	1.00	0.97	1.03	0.995

## References

[1] GBD Chronic Kidney Disease Collaboration (2020). Global, regional, and national burden of chronic kidney disease, 1990-2017: a systematic analysis for the Global Burden of Disease Study 2017. Lancet.

[2] Japanese Society of Nephrology (2019). Essential points from evidence-based clinical practice guidelines for chronic kidney disease 2018. Clin Exp Nephrol.

[3] Ortiz A, Mattace-Raso F, Soler MJ, Fouque D (2022). Ageing meets kidney disease. Clin Kidney J.

[4] Kidney Disease: Improving Global Outcomes (KDIGO) CKD Work Group (2024). KDIGO 2024 clinical practice guideline for the evaluation and management of chronic kidney disease. Kidney Int.

[5] Soysal P, Hurst C, Demurtas J (2021). Handgrip strength and health outcomes: Umbrella review of systematic reviews with meta-analyses of observational studies. J Sport Health Sci.

[6] Leong DP, Teo KK, Rangarajan S (2015). Prospective urban rural epidemiology (PURE) study investigators. Prognostic value of grip strength: findings from the prospective urban rural epidemiology (PURE) study. Lancet.

[7] Lee YL, Jin H, Lim JY, Lee SY (2021). Relationship between low handgrip strength and chronic kidney disease: KNHANES 2014-2017. J Ren Nutr.

[8] Zhang F, Wang H, Bai Y, Huang L, Zhang H (2023). Handgrip strength and all-cause mortality in patients with chronic kidney disease: an updated systematic review and meta-analysis of cohort studies. Int Urol Nephrol.

[9] He P, Ye Z, Liu M (2023). Association of handgrip strength and/or walking pace with incident chronic kidney disease: A UK biobank observational study. J Cachexia Sarcopenia Muscle.

[10] Lee SB, Kim M, Lee HJ, Kim JK (2023). Association of handgrip strength with new-onset CKD in Korean adults according to gender. Front Med (Lausanne).

[11] Japan nephrology society (2012). [Special issue: Clinical practice guidebook for diagnosis and treatment of chronic kidney disease 2012]. Nihon Jinzo Gakkai Shi.

[12] Chi JH, Lee BJ (2024). Association of relative hand grip strength with myocardial infarction and angina pectoris in the Korean population: a large-scale cross-sectional study. BMC Public Health.

[13] Lawman HG, Troiano RP, Perna FM, Wang CY, Fryar CD, Ogden CL (2016). Associations of relative handgrip strength and cardiovascular disease biomarkers in U.S. adults, 2011-2012. Am J Prev Med.

[14] Li D, Guo G, Xia L (2018). Relative handgrip strength is inversely associated with metabolic profile and metabolic disease in the general population in China. Front Physiol.

[15] (2024). Ministry of Health, Labour and Welfare: Standard questionnaire. https://www.mhlw.go.jp/seisakunitsuite/bunya/kenkou_iryou/kenkou/seikatsu/dl/hoken-program2_02.pdf.

[16] Matsuo S, Imai E, Horio M (2009). Revised equations for estimated GFR from serum creatinine in Japan. Am J Kidney Dis.

[17] Umemura S, Arima H, Arima S (2019). The Japanese Society of Hypertension Guidelines for the management of hypertension (JSH 2019). Hypertens Res.

[18] Araki E, Goto A, Kondo T (2020). Japanese clinical practice guideline for diabetes 2019. J Diabetes Investig.

[19] Kinoshita M, Yokote K, Arai H (2018). Japan Atherosclerosis Society (JAS) guidelines for prevention of atherosclerotic cardiovascular diseases 2017. J Atheroscler Thromb.

[20] Cheng Y, Liu M, Liu Y (2021). Chronic kidney disease: prevalence and association with handgrip strength in a cross-sectional study. BMC Nephrol.

[21] Bian AL, Hu HY, Rong YD, Wang J, Wang JX, Zhou XZ (2017). A study on relationship between elderly sarcopenia and inflammatory factors IL-6 and TNF-α. Eur J Med Res.

[22] Merz KE, Thurmond DC (2020). Role of skeletal muscle in insulin resistance and glucose uptake. Compr Physiol.

[23] Balakrishnan R, Thurmond DC (2022). Mechanisms by which skeletal muscle myokines ameliorate insulin resistance. Int J Mol Sci.

[24] Artunc F, Schleicher E, Weigert C, Fritsche A, Stefan N, Häring HU (2016). The impact of insulin resistance on the kidney and vasculature. Nat Rev Nephrol.

[25] Chen LK, Liu LK, Woo J (2014). Sarcopenia in Asia: consensus report of the Asian Working Group for Sarcopenia. J Am Med Dir Assoc.

[26] Wang K, Liu Q, Tang M (2023). Chronic kidney disease-induced muscle atrophy: Molecular mechanisms and promising therapies. Biochem Pharmacol.

[27] Tanimoto Y, Watanabe M, Kono R, Hirota C, Takasaki K, Kono K (2010). [Aging changes in muscle mass of Japanese]. Nihon Ronen Igakkai Zasshi.

[28] Conte C, Antonelli G, Melica ME, Tarocchi M, Romagnani P, Peired AJ (2023). Role of sex hormones in prevalent kidney diseases. Int J Mol Sci.

[29] Nishi Y, Satoh M, Nagasu H (2013). Selective estrogen receptor modulation attenuates proteinuria-induced renal tubular damage by modulating mitochondrial oxidative status. Kidney Int.

[30] Straub RH (2007). The complex role of estrogens in inflammation. Endocr Rev.

[31] Ames MK, Atkins CE, Pitt B (2019). The renin-angiotensin-aldosterone system and its suppression. J Vet Intern Med.

[32] Lu H, Lei X, Klaassen C (2006). Gender differences in renal nuclear receptors and aryl hydrocarbon receptor in 5/6 nephrectomized rats. Kidney Int.

[33] Ricardo AC, Yang W, Sha D (2019). Sex-related disparities in CKD progression. J Am Soc Nephrol.

[34] Chang PY, Chien LN, Lin YF, Wu MS, Chiu WT, Chiou HY (2016). Risk factors of gender for renal progression in patients with early chronic kidney disease. Medicine (Baltimore).

